# Contact Force-Guided versus Contact Force-Blinded Cavo-Tricuspid Isthmus Ablation for Atrial Flutter: A Systematic Review and Meta-Analysis

**DOI:** 10.3390/diseases11030098

**Published:** 2023-07-20

**Authors:** Mohamed Abuelazm, Islam Mohamed, Amith Reddy Seri, Omar Almaadawy, Basel Abdelazeem, James Robert Brašić

**Affiliations:** 1 Faculty of Medicine, Tanta University, Tanta 31527, Egypt; dr.mabuelazm@gmail.com; 2 Department of Internal Medicine, University of Missouri, Kansas City, MO 64108, USA; islam_94@hotmail.com; 3 Department of Internal Medicine, McLaren Health Care, Flint, MI 48532, USA; seriamithreddy@gmail.com (A.R.S.); baselelramly@gmail.com (B.A.); 4 Department of Internal Medicine, Michigan State University, East Lansing, MI 48823, USA; 5 Department of Internal Medicine, MedStar Health, Baltimore Internal Medicine Residency Program, Baltimore, MD 21218, USA; omaadawy@gmail.com; 6 Section of High-Resolution Brain Positron Emission Tomography Imaging, Division of Nuclear Medicine and Molecular Imaging, The Russell H. Morgan Department of Radiology and Radiological Science, The Johns Hopkins University School of Medicine, Baltimore, MD 21287, USA; 7 Department of Psychiatry, New York City Health and Hospitals/Bellevue, New York, NY 10016, USA; 8 Department of Psychiatry, New York University Grossman School of Medicine, New York University Langone Health, New York, NY 10016, USA

**Keywords:** arrhythmia, atrial fibrillation, capacitance, clinical trial, confidence interval, electrophysiology, fluoroscopy, impedance, odds ratio, radiofrequency

## Abstract

Contact force (CF) is a novel approach developed to increase the safety and efficacy of catheter ablation. However, the value of CF-sensing technology for atrial flutter (AFL) cavo-tricuspid isthmus ablation (CTIA) is inconclusive. To generate a comprehensive assessment of optimal extant data on CF for AFL, we synthesized randomized controlled trials (RCTs) and observational studies from Web of Science, SCOPUS, EMBASE, PubMed, and Cochrane until 29 November 2022, using the odds ratio (OR) for dichotomous outcomes and mean difference (MD) for continuous outcomes with a corresponding 95% confidence interval (CI). Two RCTs and three observational studies with a total of 376 patients were included in our analysis. CF-guided ablation was associated with (A) a higher rate of AFL recurrence (OR: 2.26 with 95% CI [1.05, 4.87]) and total CF (MD: 2.71 with 95% CI [1.28, 4.13]); (B) no effect on total procedure duration (MD: −2.88 with 95% CI [−7.48, 1.72]), fluoroscopy duration (MD: −0.96 with 95% CI [−2.24, 0.31]), and bidirectional isthmus block (BDIB) (OR: 1.50 with 95% CI [0.72, 3.11]); and (C) decreased radiofrequency (RF) duration (MD: −1.40 with 95% CI [−2.39, −0.41]). We conclude that although CF-guided CTIA was associated with increased AFL recurrence and total CF and reduced RF duration, it did not affect total procedure duration, fluoroscopy duration, or BDIB. Thus, CF-guided CTIA may not be the optimal intervention for AFL. These findings indicate the need for (A) providers to balance the benefits and risks of CF when utilizing precision medicine to develop treatment plans for individuals with AFL and (B) clinical trials investigating CF-guided catheter ablation for AFL to provide definitive evidence of optimal CF-sensing technology.

## 1. Introduction

Atrial flutter (AFL) is classified into typical or atypical AFL based on the cavo-tricuspid isthmus (CTI) involvement. Although typical AFL is characterized by a macro-reentrant circuit traversing the CTI, atypical AFL can arise from any region of the right or left atria and, more specifically, in areas with cardiac scar tissue but without CTI affection. Cavo-tricuspid isthmus ablation (CTIA) using radiofrequency (RF) energy is the mainstay treatment for typical AFL, with an acute procedural success rate of 95% for first-time ablation and recurrent AFL occurring only in 10% of patients over a follow-up period of four years [1]. Compared with pharmacological therapy, RF ablation showed better results in terms of rehospitalization, rhythm control, recurrence, and reported functional status [2].

CTIA is usually performed via a femoral approach under fluoroscopic guidance or using a three-dimensional mapping system. After an ablation catheter is placed at the CTI, RF energy is applied to create an ablation line from the annulus to the inferior vena cava. The contact force (CF) between the catheter electrode tip and cardiac tissue is a key determinant for procedural efficacy, defined by bidirectional CTI block. This led to the innovation of new techniques that allow the direct measurement of catheter contact, tissue impedance, and capacitance. The lesion size index (LSI) is a novel dimensionless contact force parameter that provides an accurate estimate of the lesion volume in real time by integrating contact force (grams), duration (seconds), and power (watts) [3]. This can help to guide RF ablation as disproportionate CF can lead to complications such as steam pop (SP) and cardiac perforation, while sub-optimal CF can lead to ineffective ablation lesions leading to electrical reconnection [4].

CF-guided catheter ablation was proposed to be a superior technique for atrial fibrillation (AF) ablation in comparison to standard catheter ablation (CA) [5,6]. A meta-analysis that included nine randomized controlled trials (RCTs) and twenty-six controlled observation studies (OS) revealed overall improved AF freedom, procedure duration, ablation duration, and fluoroscopy duration. However, when confined to RCTs only, CF-guided CA showed no improvement in safety or efficacy, despite observational data showing significant improvement [7]. Nevertheless, the effects of CF-guided CA on potentially fatal AFL are uncertain. Accordingly, in this systematic review and meta-analysis, we sought to compare CF-guided ablation to CF-blinded ablation in AFL patients undergoing CTIA.

## 2. Materials and Methods

This systematic review and meta-analysis was thoroughly conducted following the Preferred Reporting Items for Systematic Reviews, Meta-analysis (PRISMA) [8] (See Appendix A); and the Cochrane Handbook for Systematic Reviews of Interventions [9].

### 2.1. Data Sources and Search Strategy

Two reviewers (B.A. and M.A.) conducted a comprehensive search of the following databases until 29 November 2022 without using any search limits: PubMed, EMBASE, Web of Science (WOS), SCOPUS, and Cochrane Library. The comprehensive search terms and findings are elaborated in (Table 1).

### 2.2. Eligibility Criteria

We included RCTs and observational comparative studies with the following population intervention control outcome (PICO) criteria: population (P) as patients with AFL undergoing CTIA; intervention (I) as CF-guided ablation; control (C) as CF-blinded ablation; outcome (O) as recurrence rate of AFL. The secondary outcomes include procedural outcomes (total CF, total procedure duration, fluoroscopy duration, bidirectional isthmus block (BDIB), RF duration, and the number of lesion ablations).

The exclusion criteria involved animal studies, case reports, case series, non-randomized trials, laboratory studies, and conference abstracts.

### 2.3. Study Selection

Two reviewers (A.R.S. and O.A.) independently screened the titles and abstracts of the articles identified in the search and assessed the full-text articles for eligibility based on predefined inclusion and exclusion criteria. Any disagreement was resolved via discussion or by a third reviewer (B.A.). The included studies were reported in a Preferred Reporting Items for Systematic Reviews and Meta-Analyses (PRISMA) flow diagram [8] (Appendix A).

### 2.4. Data Extraction

Two independent investigators (A.R.S. and O.A.) extracted the summary, baseline, and outcome data from the included studies. They extracted study characteristics (country, study design, total participants, main inclusion criteria, the primary outcome, method of AFL recurrence detection, and follow-up duration); baseline characteristics (age, gender, number of patients in each group, {congestive heart failure, hypertension, age > 75, diabetes mellitus, and prior stroke or transient ischemic attack (CHA2DS-VASc) score [10]}, left ventricular ejection fraction (LVEF), and comorbidities {history of AF, hypertension (HTN), heart failure (HF), ischemic heart disease (IHD), diabetes mellitus (DM), stroke/transient ischemic attack (TIA)}; and outcomes data (AFL recurrence, total CF, total procedure duration, fluoroscopy duration, BDIB, RF duration, and number of lesion ablations). Any disagreement was resolved via discussion or by a third reviewer (B.A.).

### 2.5. Risk of Bias and Quality Assessment

Two investigators, A.R.S. and O.A., independently assessed the risk of bias in the included studies using the Cochrane Collaboration’s updated RoB 2 tool [11]. They evaluated six criteria: random sequence generation, allocation concealment, blinding of participants and personnel, blinding of outcome assessment, incomplete outcome data, and selective reporting. Additionally, A.R.S. and O.A. employed the Risk Of Bias In Non-randomized Studies—of Interventions (ROBINS-I) tool [12] to evaluate the quality of the observational studies included. Any disagreements were resolved via discussion or with the involvement of a third reviewer, B.A.

### 2.6. Statistical Analysis

The Revman software version 5.4 [13] was utilized for this meta-analysis to combine dichotomous outcomes using odds ratio (OR) and continuous outcomes using mean difference (MD), accompanied by their respective 95% confidence intervals (CI). The fixed-effects model was employed for the pooled analysis, but if substantial heterogeneity was detected, the random-effects model was used instead. Heterogeneity was assessed using the chi-square test and quantified via the I-square test. Significance for the chi-square test was set at an alpha level below 0.1, and heterogeneity was considered significant if the I-square value exceeded 50%. On significant heterogeneity, sensitivity analysis by excluding one study at a time and rerunning the analysis was conducted to investigate the source of heterogeneity. Furthermore, we conducted a subgroup analysis based on the study design. Finally, we did not investigate the publication bias by funnel plots as we included less than ten studies [14].

## 3. Results

### 3.1. Search Results and Study Selection

**Figure 1:**
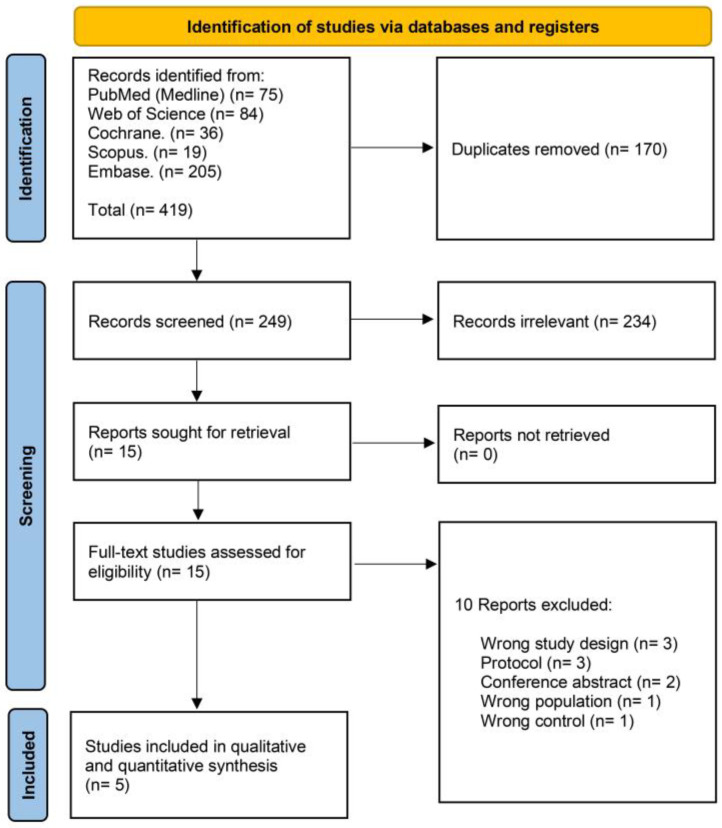
PRISMA flow chart of the screening process.

Our initial database search identified 419 records. Using COVIDence systemic review software] [15], we removed 170 duplicates and then eliminated 234 records by title and abstract screening. We then read the full text of the remaining 15 studies to finally include five studies (Figure 1).

### 3.2. Characteristics of Included Studies

We included five studies [1,15,16,17,18]: two RCTs, two prospective observational studies, and one retrospective observational study. Detailed summary characteristics of the included studies are outlined in Table 2. They were conducted in the United Kingdom, Canada, Denmark, and Australia. A total of 376 patients were included, of which 185 patients were allocated to the CF-guided group and 192 patients to the CF-blinded group. Most patients were men, including 144 (75%) men in the CF-guided group and 155 (83.7%) men in the CF-blinded. Detailed baseline characteristics of the included participants are outlined in Table 3.

It is noteworthy that the mean CF and rates of AFL recurrence varied among the included three distinct studies. In the study conducted by Begg et al. [15], we found that the total CF used in the CF-guided group was 11.4 g, accompanied by a standard deviation (SD) of 4.4. Interestingly, no cases of AFL recurrence were observed in this group, with a 0% recurrence rate at both the 3-month and 6-month checkups, mirroring results in the CF-blinded group. A comparable result was seen in Venier et al. [18], the mean CF was recorded at 13.1 g, along with an SD of 3.3. Similar to the Begg et al. study [15], the rate of AFL recurrence remained at 0%, with no cases found out of 35 subjects at both the 3-month and 6-month timepoints. Finally, Giehm-Reese et al. [1] demonstrated a mean CF of 16.7 g, with an SD of 7.5. AFL recurrence rates in this study were slightly higher, with 7 out of 66 individuals (approximately 10.6%) showing recurrence at the 3-month mark and 10 out of 58 individuals (approximately 17.2%) at the 12-month mark.

### 3.3. Risk of Bias and Quality of Evidence

**Figure 2:**
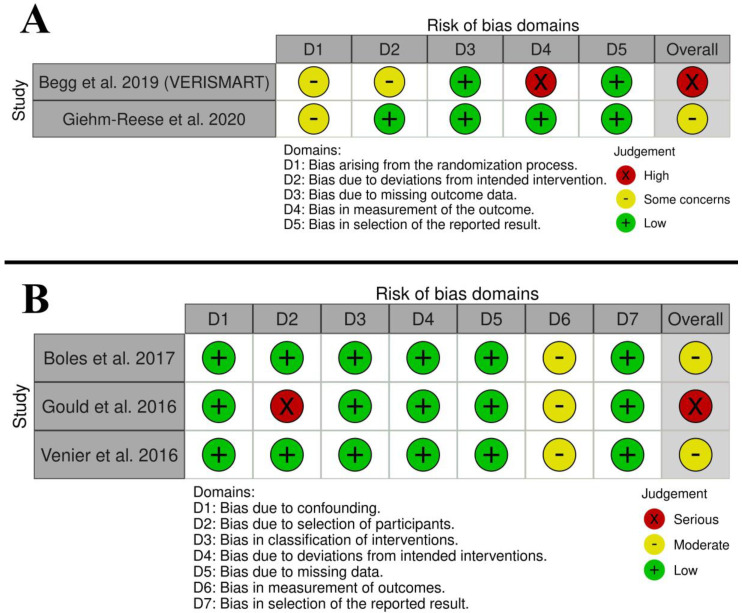
Quality assessment of risk of bias in the included studies: {(**A**) RCTs assessed by RoB 2 [1,11,15] and (**B**) observational studies assessed by ROBINS-I [12,16,17,18]}.

Begg et al. [15] associated a high overall risk of bias (RoB) with a high risk of outcome measurement bias as the authors provided no information about outcome assessor blinding, randomization, and deviation from intended interventions, while Giehm-Reese et al. [1] noted significant differences in the baseline data of the participants between both groups (Figure 2A). Also, Boles et al. [16] and Venier et al. [18] observed a moderate overall RoB, while Gould et al. [17] noted a serious overall RoB (Figure 2B).

### 3.4. Primary Outcome (AFL Recurrence)

**Figure 3:**
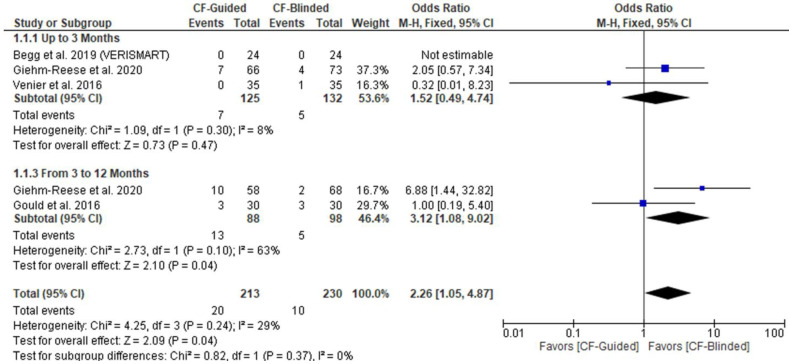
Forest plot of the primary outcome (mortality). OR: odds ratio, CI: confidence interval [1,15,17,18].

There was no difference between CF-guided and CF-blinded groups up to three months of follow up (OR: 1.52 with 95% CI [0.49, 4.74], *p* = 0.47); however, CF-guided ablation was associated with less AFL recurrence from 3 to 12 months of follow up (OR: 3.12 with 95% CI [1.08, 9.02], *p* = 0.04) (Figure 3). Pooled studies were homogenous up to three months of follow up (I^2^ = 8%, *p* = 0.3). However, pooled studies were heterogenous from 3 to 12 months of follow up (I^2^ = 63%, *p* = 0.1). Hence, we used the random-effect model yielding no difference between both groups (OR: 2.69 with 95% CI [0.40, 17.99], *p* = 0.31). The test of subgroup difference based on the study design was not significant (*p* = 0.30) for up to three months (Appendix B).

### 3.5. Secondary Outcomes

**Figure 4:**
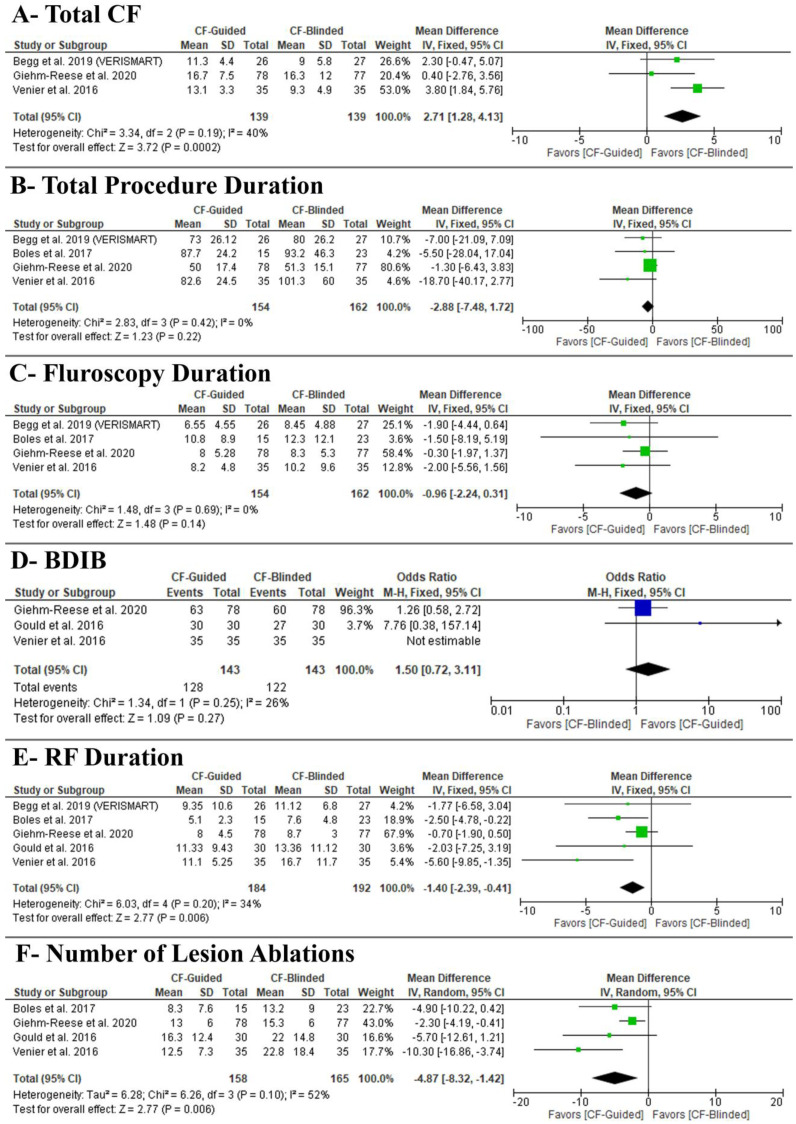
Forest plot of the secondary outcomes. OR: odds ratio, MD: mean difference, CI: confidence interval [1,15,16,17,18].

CF-guided ablation was associated with a higher total CF (MD: 2.71 with 95% CI [1.28, 4.13], *p* = 0.0002) (Figure 4A); no effect on total procedure duration (MD: −2.88 with 95% CI [−7.48, 1.72], *p* = 0.22) (Figure 4B), fluoroscopy duration (MD: −0.96 with 95% CI [−2.24, 0.31], *p* = 0.14) (Figure 4C), and BDIB (OR: 1.50 with 95% CI [0.72, 3.11], *p* = 0.27) (Figure 4D). However, CF-guided ablation was associated with decreased RF duration (MD: −1.40 with 95% CI [−2.39, −0.41], *p* = 0.006) (Figure 4E) and the number of lesion ablations (MD: −4.87 with 95% CI [−8.32, −1.42], *p* = 0.006) (Figure 4F).

Pooled studies were homogenous in total CF (I^2^ = 40%, *p* = 0.19), total procedure duration (I^2^ = 0%, *p* = 0.42), fluoroscopy duration (I^2^ = 0%, *p* = 0.69), BDIB (I^2^ = 26%, *p* = 0.25), and RF duration (I^2^ = 34%, *p* = 0.20). However, pooled studies were heterogenous in the number of lesion ablations (I^2^ = 52%, *p* = 0.1). We conducted a sensitivity analysis to investigate the source of heterogeneity, and it was best resolved after excluding Giehm-Reese et al. (I^2^ = 0%, *p* = 0.43) or Venier et al. 2016 (I^2^ = 0%, *p* = 0.46) with stable results favoring CF-guided ablation.

The test of subgroup difference based on the study design was not significant in total CF (*p* = 0.11), total procedure duration (*p* = 0.32), fluoroscopy duration (*p* = 0.81), and BDIB (*p* = 0.25) (Appendix B). However, it was significant in RF duration (*p* = 0.09) and the number of lesion ablations (*p* = 0.07). In RF duration, only observational studies; prospective (MD: −4.18 with 95% CI [−7.47, −0.88], *p* = 0.01) and retrospective (MD: −2.50 with 95% CI [−4.78, −0.22], *p* = 0.03) favored contact force-guided group. However, RCTs found no difference between both groups (MD: −0.76 with 95% [−1.93, 0.40] CI], *p* = 0.20). In the number of lesion ablations, RCTs (MD: −2.30 with 95% CI [−4.19, −0.41], *p* = 0.02) and prospective studies (MD: −8.12 with 95% CI [−12.88, −3.36], *p* = 0.0008) favored contact force-guided group. However, retrospective studies found no difference between both groups (MD −4.90 with 95% [−10.22, 0.42], *p* = 0.07) (Appendix B).

## 4. Discussion

Based on the results of two RCTs, two prospective studies, and one retrospective study with a total of 376 patients, we conclude that CF-guided ablation is associated with (A) a higher incidence of AFL recurrence and total CF with CF-guided ablation, (B) no effect on the total procedure duration, fluoroscopy duration, or BDIB, and (C) shorter RF duration and fewer ablations per lesion. Thus, we identify characteristics of CF ablation that must be weighed by providers considering the risks and benefits of available interventions.

Our systematic review and meta-analysis until 29 November 2022, to compare CF-guided ablation versus CF-blinded ablation for AFL, utilized more selective criteria to detect key features of the literature that are not identified in another systemic review and meta-analysis until June 2022 [19]. Furthermore, in our study, we included contact force alone as the primary parameter of interest. Previous analysis by Pang et al. [19] included three different contact parameters: CF, electrical coupling index (ECI), and ablation index (AI). The findings indicated that the impact of all three parameters was comparable and did not significantly contribute to the inter-group differences, except for that on fluoroscopy time [19]. In their included studies, a study involving AI showed a significant reduction in fluoroscopy time among the intervention group [20]. However, the results from the other subgroups and the overall analysis did not show a statistically significant difference [19]. Our systematic review and meta-analysis provide a more subtle quality assessment by utilizing the state-of-the-art tools (RoB 2 [11] and ROBINS-1 [12]) instead of the older tools (QUADAS-2 [21] and the Newcastle-Ottawa Scale (NOS) [22] utilized by the other systematic review and meta-analysis [19]. 

CTI ablation is one of the most performed ablation procedures with a low recurrence rate [2]. AFL ablation is already a highly effective and safe procedure, but several technological and methodological developments have proposed incremental improvements in efficiency without compromising safety and effectiveness. It has been proposed that a CF-guided ablation of AFL is a novel technique with the potential to reduce total RF delivery time, the time to achieve BDIB, as well as recurrence of AFL after CF ablation [15]. However, in the absence of established guidelines, additional efforts are needed to combine available data and perform pooled analysis of available studies to reach clinically applicable conclusions. 

Despite the achievable endpoint of BDIB, a significant proportion of patients experience conduction recurrence via CTI, which can lead to recurrent arrhythmias. The ability to achieve a durable conduction block depends on the ability to obtain a stable, contiguous set of transmural lesions. This depends on various factors such as tissue depth, ablation electrode size, the temperature at the electrode–tissue interface, RF duration, and electrode tip tissue CF [3]. Recently, the development of CF-sensing catheters has contributed to a better understanding of the electrode tip–tissue CF relationship and subsequent lesion formation. It also enhances the contact between the electrode and tissue during RF catheter ablation, which can significantly improve procedure parameters, leading to considerable reductions in procedure duration and fluoroscopy exposure, without elevating the risk of immediate complications [23]. Considerable reductions in the occurrence of acute pulmonary vein (PV) reconnection and resting conduction have been noted during AF ablation procedures utilizing live CF data for PV Isolation. Furthermore, incorporating CF sensors for PV isolation not only decreases procedure duration but also minimizes the requirement for supplementary ablation, leading to improved long-term outcomes [24,25].

While there is growing evidence to support the significant impact of CF in assessing the effectiveness of lesions and enhancing the success rate of AF ablation, the exact role of CF guidance in CTI ablation of AFL remains uncertain. CTI is highly heterogeneous, with tissue thickness decreasing from the annulus to the vena cava, as well as the presence of multiple prominences, including ridges, pouches, or pectinate muscles. All these factors can affect the ability to create adequate ablation lesions and thus can benefit most from CF detection techniques. The results of our study indicate that CF-guided CTIA led to a significant reduction in lesion ablations but a higher recurrence rate of AFL. Based on a subgroup analysis, the recurrence rate of AFL in the first three months was not significantly different between CF-guided and CF-blinded groups. In contrast, the overall rate of AFL recurrence, as well as the rate of AFL recurrence from 3 to 12 months was significantly higher in the CF-guided group than in the CF-blinded group. This was mainly weighted by Giehm-Reese et al. [1], whose participants were older and had more comorbidities, including IHD requiring percutaneous cardiac intervention or coronary artery bypass graft as well as HTN [26]. CF-guided patients may experience a higher recurrence rate due to this overestimation.

Moreover, using contact sensing technology may assist in delivering effective ablation lesions. The ideal ablation lesion would cover the entire thickness of the myocardium with minimal collateral damage to surrounding tissue and without the generation of a “steam pop,” the audible sound produced by an intramyocardial explosion when tissue temperature reaches 100 °C, resulting in gas formation [27]. Due to its association with cardiac perforation and ventricular septal defect, it is a potentially severe complication of radiofrequency ablation [28]. Therefore, the goal is to provide sufficient CF between the catheter and tissue to provide sufficient RF energy to prevent AFL recurrence but also not to cause perforation or steam pops. Frances et al. [29] and Venier et al. [18] suggested that there is an inverse correlation between RF duration and the percentage of lesions requiring greater than 10 g of CF per procedure. With an average CF of less than 10 g per procedure, the RF delivery time was significantly reduced. Furthermore, several studies have demonstrated that lesions with an average CF of <10 g have a higher risk of re-conduction after AF ablation [29]. Accordingly, 10 g might be the minimum target CF required along the CTIA to reduce the RF, fluoroscopy, and total procedure duration. CF-guided ablation was associated with a higher total CF (MD: 2.71 with 95% CI 175 [1.28, 4.13], *p* = 0.0002) (Figure 4A). In both the CF-guided and CF-blinded interventions, CF-guided catheters were used. In both groups, contact force was recorded, but it was blinded to the operators in the CF-blinded group. Our study showed that the CF-guided group had a higher total CF. Excessive CF may result in complications like cardiac perforation. Achieving bidirectional block with lesser CF may be preferred to avoid such complications. Our study highlights and supports this finding since the CF-guided group achieved adequate contact force with a significant reduction in RF duration; however, we also found no difference in fluoroscopy and total procedure duration.

Radiation exposure during conventional transcatheter ablation procedures can have significant health effects, both deterministic and stochastic. Deterministic effects, such as radiation-induced skin burns, acute radiation syndrome, cataracts, sterility, and tumor necrosis, occur when a specific level of ionizing radiation exposure is reached. Stochastic effects, on the other hand, are random and probabilistic, with an extremely rare occurrence being the development of cancer in irradiated organs or tissues [30]. These effects emphasize the importance of minimizing radiation exposure whenever possible. Our study focuses on the importance of CF catheters in addressing these concerns. CF catheters facilitate improved contact and the creation of adequate ablation lesions, resulting in reduced procedure times. This reduction in procedure time is attributed to the effective and stable contact between the catheter tip and the tissue, which is crucial for both mapping and lesion formation during cardiac ablation procedures [31]. 

By maintaining consistent and adequate contact, CF catheters can minimize the need for repeat ablations or adjustments, leading to reduced procedure time. Inadequate contact force can result in incomplete or ineffective lesion formation, leading to the need for additional ablations. CF catheters aid in achieving optimal contact force, allowing for efficient lesion formation in a single application. This efficiency reduces the number of ablations required, thereby saving time during the procedure [32]. Furthermore, CF catheters not only provide a therapeutic approach to arrhythmias but also serve as a tool for accurately characterizing the arrhythmic substrate [33]. By providing precise and reliable contact force information, CF catheters enable clinicians to deliver optimal therapy while minimizing unnecessary energy delivery and the overall duration of the procedure, thereby reducing the need for extensive fluoroscopy time.

In light of these considerations, efforts have been made to reduce radiation exposure in electrophysiology. A study investigated contact force-controlled zero-fluoroscopy catheter ablation for right and left-sided arrhythmias, achieving a procedural success rate of 97% with minimal complications [33]. Additionally, a study from Italy evaluated physicians’ awareness of radiation effects via questionnaires. The findings demonstrated satisfactory awareness but recommended further improvement [34]. It also emphasized that the awareness of radiation risks is essential for fostering a culture of respect for radiation hazards and a commitment to minimizing exposure while maximizing protection [34].

Regarding complications, adverse events were not extensively reported in the studies analyzed. In the study by Venier et al. [18], two instances of steam pops occurred in the CF-blinded group, but they had no clinical consequences. Gould et al. [17] reported a minor complication of acute groin bleeding in one patient, which resolved with rest and pressure. In the Giehm-Reese study [1], complications were reported in six patients, including groin hematomas, resulting in a delayed discharge for some patients. However, none of the hematomas required blood transfusion or surgery, with the occurrence of audible steam pops comparable between both groups. Finally, Begg et al. [15] reported an overall complication rate was 2%, with one patient experiencing a transient ischemic attack and another patient requiring treatment for ventricular fibrillation. 

### 4.1. Limitations

First, the pooled analysis presented in this systematic review and meta-analysis is derived from two RCTs, two prospective studies, and one retrospective study, including a total of 376 patients, which is a small number that may have adversely affected our study’s power. Second, the inclusion of prospective and retrospective studies increased the number of patients included in this study but also increased the RoB, particularly selection bias, since the investigators were not blinded. In addition, two RCTs [1,15] included patients who were slightly older in the intervention group, and more of them were women with higher CHA2DS2-VASc scores than the control group. Despite being attributed to chance, these differences may have affected the outcomes between intervention groups. Third, the study conducted by Giehm-Reese et al. [1] was initially powered to measure re-conduction after three months but was further extended to measure recurrent arrhythmia after 12 months. 

Fourth, although theoretically, CF guidance may reduce steam pops, we acknowledge that the lack of data is a limitation of our study. However, it is worth noting that Venier et al., 2016 [18], reported no major complications, despite two steam pops occurring in the CF-blinded group, which had no clinical consequences [18]. Similarly, Giehm-Reese et al. [1] found that the number of patients with audible steam pops was similar in both the CF-blinded and guided groups. Fifth, no reports of scar size were present in the RCTs included in our study. According to Begg et al., 2019 [15], CF-guided and CF-blinded techniques produced similar ablation lesions, with 33 mm being the approximate length with no significant differences between the different techniques. The results of the four RCTs included in this review demonstrated that CF-guided ablation led to a reduction in the number of lesions required to terminate AFL. Fifth, we did not investigate other parameters, including ECI or AI, which can show different findings; however, current data imply a similarity between them and CF [19]. Future investigations can be enhanced by specific measurements of scar size. Finally, there was also a disparity between the expertise of operators in CF-guided ablation across the different studies, and this could have affected their results, as CTIA is a highly precise procedure that requires highly experienced operators to provide consistent results. Also, operators may unconsciously place more ablation lesions in CF-blinded groups to promote CF-guided ablation.

### 4.2. Future Research Implications

We reported that the AFL rate of recurrence was higher in the CF-guided group; however, AFL recurrence rates may fluctuate, and a short-term follow up might not accurately reflect the true rates. Thus, conducting long-term follow ups can offer a more comprehensive view of recurrence rates, providing insight into the enduring effectiveness of CF-guided procedures and identifying late recurrences. Also, standardizing ablation procedure techniques, including catheter position, energy delivery parameters, lesion creation protocol, and post-procedure management, can also help minimize variability, ensure consistency, and enable the accurate evaluation of CF guidance’s impact on AFL recurrence rates. Finally, operator proficiency also significantly impacts procedure outcomes, necessitating the inclusion of experienced operators in future studies. Providing standardized training and certification programs can further enhance consistency, reduce variations, and ultimately impact AFL recurrence rates. Clinical trials are needed to investigate CF-guided catheter ablation for AFL to provide definitive evidence of optimal CF-sensing technology.

## 5. Conclusions

CF-guided CTIA is associated with (A) increased risk of AFL recurrence and total CF, (B) no effect on the fluoroscopy duration, the total procedure duration, or the BDIB, and (C) reduced RF duration and the number of lesion ablations. The clinical application of CF technology in the CTIA of AFL requires further rigorous RCTs as currently available evidence is mainly derived from two small single-center RCTs and observational studies. The potentially fatal outcomes of AFL in people with heart transplantations [33] and other procedures emphasize the need for definitive studies of CF-blinded CTIA and CF-guided CTIA. This study suggests that CF-guided CTIA may not be the optimal intervention for AFL. Providers must carefully consider the adverse and beneficial effects of interventions when developing treatment plans to apply precision medicine for individuals with AFL.

## Figures and Tables

**Table 1:** Search terms and results in different databases.

Database	Search Terms	Search Field	Search Results
PubMed	(“Contact force” OR contact force-sens* OR “Cavo-tricuspid isthmus ablation” OR CTIA) AND (“Atrial flutter” OR AFL OR “Auricular Flutter”)	All Fields	75
Cochrane	(“Contact force” OR contact force-sens* OR “Cavo-tricuspid isthmus ablation” OR CTIA) AND (“Atrial flutter” OR AFL OR “Auricular Flutter”)	All Fields	36
WOS	(“Contact force” OR contact force-sens* OR “Cavo-tricuspid isthmus ablation” OR CTIA) AND (“Atrial flutter” OR AFL OR “Auricular Flutter”)	All Fields	84
SCOPUS	TITLE-ABS-KEY ((“Contact force” OR contact AND force-sens* OR “Cavo-tricuspid isthmus ablation” OR ctia ) AND (“Atrial flutter” OR afl OR “Auricular Flutter”))	Title, Abstract, Keyword	19
EMBASE	#3. #1 AND #2 #2. ‘atrial flutter’:ti, ab, kw OR afl:ti, ab, kw OR ‘auricular flutter’:ti, ab, kw #1. ‘contact force’:ti, ab, kw OR ‘contact force-sens*’:ti, ab, kw OR ‘cavo-tricuspid isthmus ablation’:ti, ab, kw OR ctia:ti, ab, kw	All Fields	205

**Table 2:** Characteristics of the included studies.

Study ID	Study Design	C	T	Main Inclusion Criteria	Method of AFL Recurrence Detection	Primary Outcome	Follow-Up Duration	Ablation Catheter	CF Target (g)	Mean CF (g)
Begg et al. 2019 (VERISMART) [15]	Multicenter RCT	UK	53	Persistent or paroxysmal AFL.	Seven days of ECG monitoring	Time to BDIB	Six months	Thermocool Smart Touch	5–40	N/A
Boles et al. 2017 [16]	Retrospective single-center observational study	CA	38	Persistent or paroxysmal AFL	N/A	Complete BDIB	N/A	TactiCath Quartz (CF) CoolFlex (non-CF)	10–30	13.9
Giehm-Reese et al. 2020 [1]	Multicenter double-blinded superiority RCT	DK	155	Typical AFL undergoing first-time CTIA	Five days Holter ECG at one month and invasive EPS study at three months	Recurrent isthmus conduction measured with invasive EPS three months after ablation	Three months	TactiCathTM Quartz	10–30	16.3
Gould et al. 2016 [17]	Prospective single-center observational study with retrospective historical control	AU	60	Typical AFL	ECG and Holter monitor	BDIB	12 months	Tacti-Cath, Quartz (CF) 8 mm F-Curve Biosense Webster Thermocouple catheter (non-CF)	10–40	17
Venier et al. 2016 [18]	Prospective single-center observational study	CA	70	Typical AFL undergoing first-time CTIA	24 h Holter monitor and 12-lead ECG	BDIB	Six months	Thermocool Smart Touch	10–25	11.5

RCT: randomized controlled trial, C: country, T: total, UK: United Kingdom, CA: Canada, DK: Denmark, AU: Australia, AFL: atrial flutter, CTIA: cavotricuspid isthmus ablation, ECG: electrocardiogram, EPS: electrophysiology study, BDIB: bidirectional isthmus block, CF: contact force, N/A: not available.

**Table 3:** Baseline characteristics of the participants.

Study ID	Number of Patients in Each Group		Age (Years) Mean (SD)		Gender (Male) *N* (%)		CHA2DS-VASc Score Mean (SD)		LVEF (%) Mean (SD)		AFL Duration (Months) Mean (SD)		Comorbidities *N* (%)											
	CFG	CFB	CFG	CFB	CFG	CFB	CFG	CFB	CFG	CFB	CFG	CFB	AF		HTN		HF		DM		IHD		Stroke/ TIA	
													CFG	CFB	CFG	CFB	CFG	CFB	CFG	CFB	CFG	CFB	CFG	CFB
Begg et al. 2019 (VERISMART) [15]	26	27	62.7 (21.2)	65.3 (16.5)	24, (923)	21 (77.8)	2 (1.7)	1.9 (1.5)	N/A	N/A	9 (9.8)	17 (24.4)	N/A	N/A	12 (46.2)	11 (40.7)	6 (23.1)	4 (14.8)	7 (26.9)	5 (8.5)	8 (30.8)	3 (11.1)	(1 3.8)	0
Boles et al. 2017 [16]	15	23	69 (7.9)	66.3 (10.4)	10 (66.6)	16 (69.6)	2.6 (1.6)	2.5, (1.6)	53.5 (15.9)	51.4 (22)	N/A	N/A	N/A	N/A	10 (66.6)	14 (61)	N/A	N/A	5 (33.3)	5 (21.7)	5 (33.3)	7 (30.4)	N/A	N/A
Giehm-Reese et al. 2020 [1]	79	77	69.3 (9.8)	65.7 (12.1)	55 (70)	65 (84)	3 (1.5)	2 (1.5)	53.3 (11.3)	55 (11.3)	N/A	N/A	4 (5)	2 (3)	46 (58)	36 (47)	26 (33)	23 (30)	13 (16)	18 (23)	19 (24)	10 (13)	8 (10)	8 (10)
Gould et al. 2016 [17]	30	30	64 (8)	64 (11)	23 (76.7)	24 (80)	N/A	N/A	57 (6)	56 (7)	N/A	N/A	N/A	N/A	N/A	N/A	N/A	N/A	N/A	N/a	N/A	N/A	N/A	N/A
Venier et al. 2016 [18]	35	35	63.9 (12.4)	61.5 (9.2)	32 (91)	29 (83)	1.2 (0.9)	0.8 (0.7)	55 (11.1)	56.4 (7.2)	N/A	N/A	17 (49)	20 (57)	15 (43)	18 (51)	N/A	N/A	10 (29)	9 (26)	7 (20)	5 (14)	N/A	N/A

CFB: contact force-blinded, CFG: contact force-guided, N/A: not available, LVEF: left ventricular ejection fraction, HTN: hypertension, HF: heart failure, DM: diabetes mellitus, IHD: ischemic heart disease, TIA: transient ischemic attack, SD: standard deviation, *N*: number.

**Figure A1:**
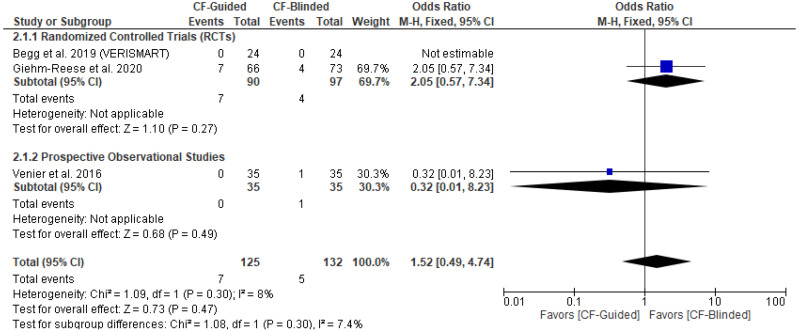
Forest plot of AFL recurrence for up to 3 months subgrouped by the study design [1,15,18].

**Figure A2:**
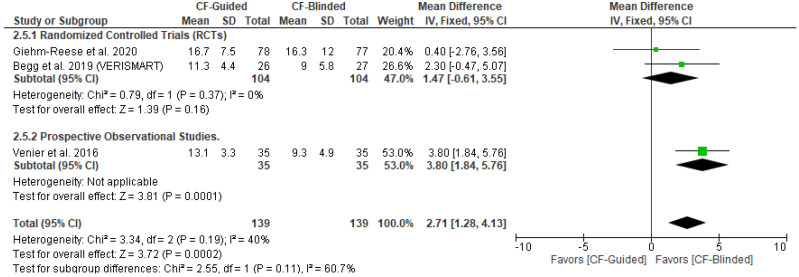
Forest plot of total CF subgrouped by the study design [1,15,18].

**Figure A3:**
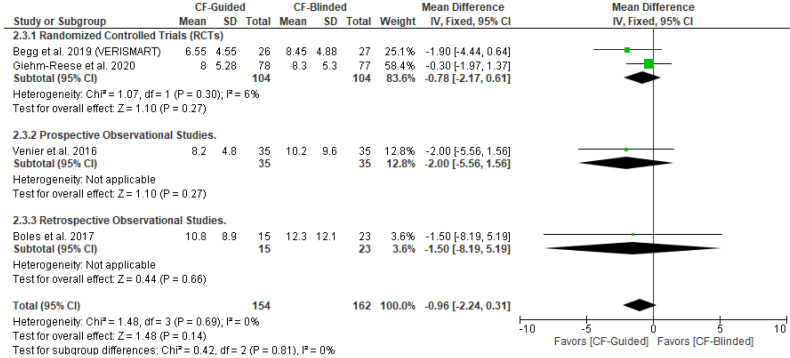
Forest plot of total procedure duration subgrouped by the study design [1,15,16,18].

**Figure A4:**
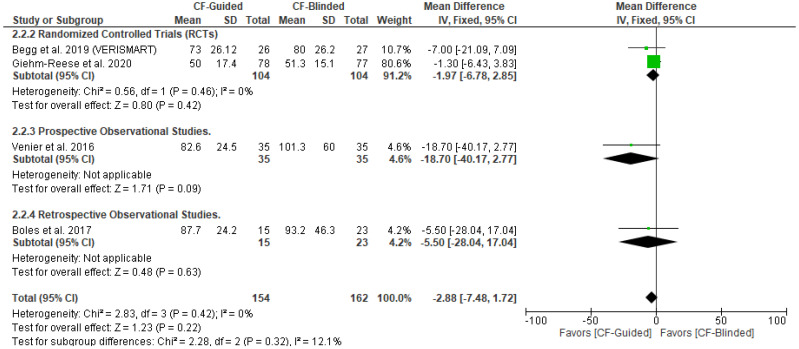
Forest plot of fluoroscopy duration subgrouped by the study design [1,15,16,18].

**Figure A5:**
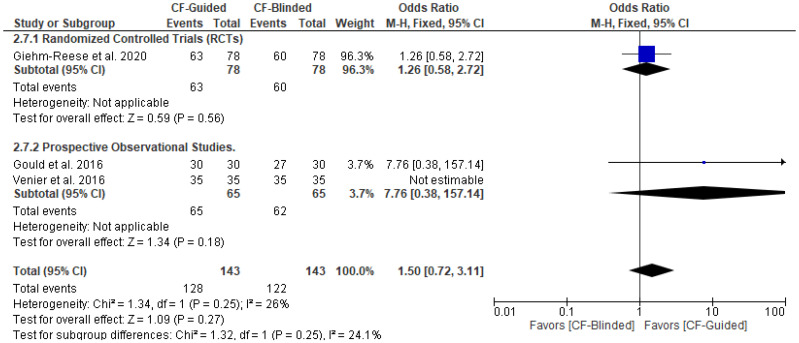
Forest plot of BDIB subgrouped by the study design [1,17,18].

**Figure A6:**
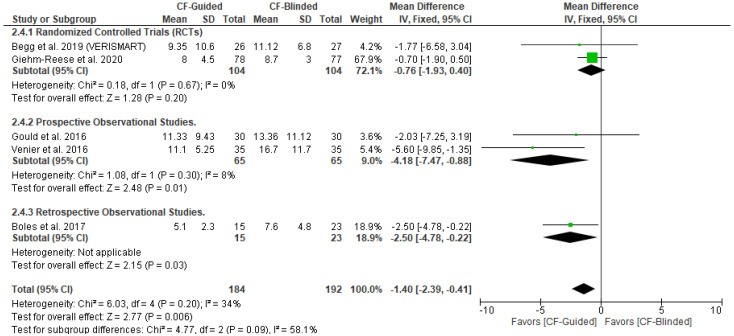
Forest plot of radiofrequency duration subgrouped by the study design [1,15,16,17,18].

**Figure A7:**
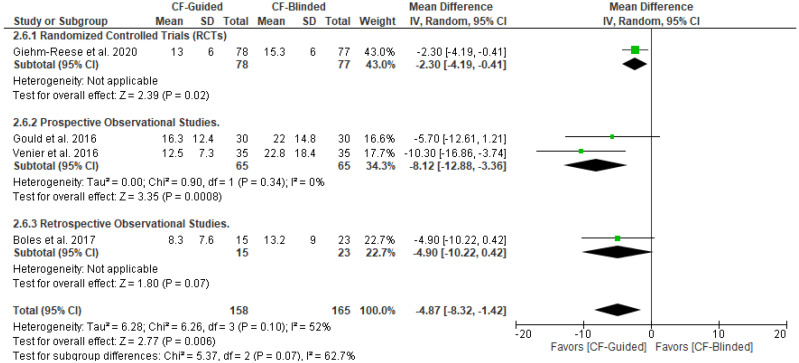
Forest plot of the number of lesion ablations subgrouped by the study design [1,16,17,18].

## Data Availability

All data are included in the manuscript.

## Abbreviations

AF	Atrial fibrillation
AFL	Atrial flutter
BDIB	Bidirectional isthmus block
CF	Contact force
CHA2DS-VASc	Congestive heart failure, hypertension, age > 75, diabetes mellitus, and prior stroke or transient ischemic attack [10]
CI	Confidence interval, the lower and upper limits of significance
CTI	Cavo-tricuspid isthmus
CTIA	Cavo-tricuspid isthmus ablation
DM	Diabetes mellitus
ECG	Electrocardiogram
EPS	Electrophysiology study
HF	Heart failure
HTN	Hypertension
ICH	Intracranial hemorrhage
ID	Identification
IHD	Ischemic heart disease
LSI	Lesion size index
LVEF	Left ventricular ejection fraction
MD	Mean difference
N	Number
N/A	Not available
NOS	Newcastle–Ottawa Scale [23]
OR	Odds ratio
OS	Observation study
*p*	Probability
PICO	Population intervention control outcome
PRISMA	Preferred Reporting Items for Systematic Reviews and Meta-Analyses [8]
PV	Pulmonary vein
QUADAS	Quality Assessment of Diagnostic Accuracy Studies [22]
RCT	Randomized controlled trial
RF	Radiofrequency
RoB	Risk of bias
RoB 2	Risk of bias 2 [11]
ROBINS-1	Risk Of Bias In Non-randomized Studies—of Interventions [12]
SD	Standard deviation
SP	Steam pop
TIA	Transient ischemic attack
WOS	Web of Science

## PRISMA 2020 Checklist [8]

**Table A1:** PRISMA 2020 Checklist.

Section and Topic	Item #	Checklist Item	Location Where Item Is Reported
**Title**			
Title	1	Identify the report as a systematic review.	Page 1 line 2
**Abstract**			
Abstract	2	See the PRISMA 2020 for Abstracts checklist.	Page 1
**Introduction**			
Rationale	3	Describe the rationale for the review in the context of existing knowledge.	Page 2
Objectives	4	Provide an explicit statement of the objective(s) or question(s) the review addresses.	page 2
**Methods**			
Eligibility criteria	5	Specify the inclusion and exclusion criteria for the review and how studies were grouped for the syntheses.	Page 3 Section 2.2
Information sources	6	Specify all databases, registers, websites, organizations, reference lists, and other sources searched or consulted to identify studies. Specify the date when each source was last searched or consulted.	Page 3 Section 2.1
Search strategy	7	Present the full search strategies for all databases, registers, and websites, including any filters and limits used.	Page 2,3 Table 1
Selection process	8	Specify the methods used to decide whether a study met the inclusion criteria of the review, including how many reviewers screened each record and each report retrieved, whether they worked independently, and if applicable, details of automation tools used in the process.	Page 3 Section 2.3
Data collection process	9	Specify the methods used to collect data from reports, including how many reviewers collected data from each report, whether they worked independently, any processes for obtaining or confirming data from study investigators, and if applicable, details of automation tools used in the process.	Page 3 Section 2.3
Data items	10a	List and define all outcomes for which data were sought. Specify whether all results that were compatible with each outcome domain in each study were sought (e.g., for all measures, time points, analyses), and if not, the methods used to decide which results to collect.	Page 3 Section 2.4
	10b	List and define all other variables for which data were sought (e.g., participant and intervention characteristics, funding sources). Describe any assumptions made about any missing or unclear information.	Page 3 Section 2.4
Study risk of bias assessment	11	Specify the methods used to assess the risk of bias in the included studies, including details of the tool(s) used, how many reviewers assessed each study and whether they worked independently, and if applicable, details of automation tools used in the process.	Page 3 Section 2.5
Effect measures	12	Specify for each outcome the effect measure(s) (e.g., risk ratio, mean difference) used in the synthesis or presentation of results.	Page 4 Section 2.6
Synthesis methods	13a	Describe the processes used to decide which studies were eligible for each synthesis (e.g., tabulating the study intervention characteristics and comparing against the planned groups for each synthesis (item #5)).	Page 4 Section 2.6
	13b	Describe any methods required to prepare the data for presentation or synthesis, such as handling of missing summary statistics, or data conversions.	Page 4 Section 2.6
	13c	Describe any methods used to tabulate or visually display the results of individual studies and syntheses.	Page 4 Section 2.6
	13d	Describe any methods used to synthesize results and provide a rationale for the choice(s). If meta-analysis was performed, describe the model(s), method(s) to identify the presence and extent of statistical heterogeneity, and software package(s) used.	Page 4 Section 2.6
	13e	Describe any methods used to explore possible causes of heterogeneity among study results (e.g., subgroup analysis, meta-regression).	Not applicable
	13f	Describe any sensitivity analyses conducted to assess the robustness of the synthesized results.	Not applicable
Reporting bias assessment	14	Describe any methods used to assess the risk of bias due to missing results in a synthesis (arising from reporting biases).	Page 3 Section 2.5
Certainty assessment	15	Describe any methods used to assess certainty (or confidence) in the body of evidence for an outcome.	Page 4 Section 2.6
**Results**			
Study selection	16a	Describe the results of the search and selection process, from the number of records identified in the search to the number of studies included in the review, ideally using a flow diagram.	Page 4 Section 3.1
	16b	Cite studies that might appear to meet the inclusion criteria, but which were excluded, and explain why they were excluded.	Not applicable
Study characteristics	17	Cite each included study and present its characteristics.	Page 5 Section 3.2
Risk of bias in studies	18	Present assessments of risk of bias for each included study.	Page 8 Section 3.3
Results of individual studies	19	For all outcomes, present, for each study: (a) summary statistics for each group (where appropriate) and (b) an effect estimate and its precision (e.g., confidence/credible interval), ideally using structured tables or plots.	Pages 9, 10 Section 3.4 and Section 3.5
Results of syntheses	20a	For each synthesis, briefly summarize the characteristics and risk of bias among contributing studies.	Page 5 Section 3.2 and Section 3.3
	20b	Present results of all statistical syntheses conducted. If meta-analysis was conducted, present for each the summary estimate and its precision (e.g., confidence/credible interval) and measures of statistical heterogeneity. If comparing groups, describe the direction of the effect.	Pages 9, 10
	20c	Present results of all investigations of possible causes of heterogeneity among study results.	Not applicable
	20d	Present results of all sensitivity analyses conducted to assess the robustness of the synthesized results.	Not applicable
Reporting biases	21	Present assessments of risk of bias due to missing results (arising from reporting biases) for each synthesis assessed.	Page 8 Section 3.3
Certainty of evidence	22	Present assessments of certainty (or confidence) in the body of evidence for each outcome assessed.	Not available
**Discussion**			
Discussion	23a	Provide a general interpretation of the results in the context of other evidence.	Page 11
	23b	Discuss any limitations of the evidence included in the review.	Page 12
	23c	Discuss any limitations of the review processes used.	Page 12
	23d	Discuss the implications of the results for practice, policy, and future research.	Page 12
**Other Information**			
Registration and protocol	24a	Provide registration information for the review, including the register name and registration number, or state that the review was not registered.	Not available
	24b	Indicate where the review protocol can be accessed, or state that a protocol was not prepared.	Not available
	24c	Describe and explain any amendments to the information provided at registration or in the protocol.	Not available
Support	25	Describe sources of financial or non-financial support for the review, and the role of the funders or sponsors in the review.	Page 12
Competing interests	26	Declare any competing interests of review authors.	Page 12
Availability of data, code, and other materials	27	Report which of the following are publicly available and where they can be found: template data collection forms; data extracted from included studies; data used for all analyses; analytic code; any other materials used in the review.	Page 12
